# Social Network Analysis and Qualitative Interviews for Assessing Geographic Characteristics of Tourism Business Networks

**DOI:** 10.1371/journal.pone.0156028

**Published:** 2016-06-03

**Authors:** Ilan Kelman, Tobias Luthe, Romano Wyss, Silje H. Tørnblad, Yvette Evers, Marina Martin Curran, Richard J. Williams, Eric L. Berlow

**Affiliations:** 1 Institute for Global Health and Institute for Risk & Disaster Reduction, University College London, London, United Kingdom; 2 University of Agder, Kristiansand, Norway; 3 Department Living Environment, University of Applied Sciences HTW Chur, Chur, Switzerland; 4 MonViso Institute, Bozen/Ostana, Italy; 5 Laboratory on Human-Environment Relations in Urban Systems (HERUS), EPFL, Lausanne, Switzerland; 6 ARENA Centre for European Studies, University of Oslo, Oslo, Norway; 7 The Forest Trust,‎ Nyon, Switzerland; 8 Business School Lausanne, Chavannes, Switzerland; 9 Vibrant Data Inc., San Francisco, United States of America; Université Toulouse 1 Capitole, FRANCE

## Abstract

This study integrates quantitative social network analysis (SNA) and qualitative interviews for understanding tourism business links in isolated communities through analysing spatial characteristics. Two case studies are used, the Surselva-Gotthard region in the Swiss Alps and Longyearbyen in the Arctic archipelago of Svalbard, to test the spatial characteristics of physical proximity, isolation, and smallness for understanding tourism business links. In the larger Surselva-Gotthard region, we found a strong relationship between geographic separation of the three communities on compartmentalization of the collaboration network. A small set of businesses played a central role in steering collaborative decisions for this community, while a group of structurally ‘peripheral’ actors were less influential. By contrast, the business community in Svalbard showed compartmentalization that was independent of geographic distance between actors. Within towns of similar size and governance scale, Svalbard is more compartmentalized, and those compartments are not driven by geographic separation of the collaboration clusters. This compartmentalization in Svalbard was reflected in a lower density of formal business collaboration ties compared to the communities of the Alps. We infer that the difference is due to Svalbard having higher cultural diversity and population turnover than the Alps communities. We propose that integrating quantitative network analysis from simple surveys with qualitative interviews targeted from the network results is an efficient general approach to identify regionally specific constraints and opportunities for effective governance.

## Introduction

Social Network Analysis (SNA) is a technique allowing the systematic quantitative and qualitative analysis of the links amongst actors in various contexts [1], assisting the understanding of how the system in which those actors operate is able to function [2]. From its origins in sociology, it has expanded across disciplines including tourism research [3] [4].

In this context, SNA reveals if and how tourism business actors are linked within a location or within a sector by specifying the concentration of links in certain parts of the network and the number of links amongst specific actors or sub-groups. Links can be of various types, referring to, for example, direct cooperation to support tourists, information exchange, financial ties such as joint suppliers, or common ownership. [3], [4] and [5] use SNA to analyse how the tourism industry in the Swiss Alps deals with external pressures, notably climate change. Tourism business networks comprising formal and informal professional collaborative links in different destinations can be compared to glean insights into how the businesses address social and environmental changes in tandem.

These SNAs do not always fully identify a destination’s entire spatial characteristics, even though the spatial characteristics influence tourism business operations as shown by the literature from, for instance, island tourism [6], [7] and mountain tourism [8], [9]. Since so many factors affect how businesses operate, a single method such as quantitative SNA or qualitative interviews could only reveal so much [10]. Examining these limitations can provide insight into methodological combinations to build on each method’s strengths (see also [11]).

This study uses quantitative SNA and qualitative interviews, combining both methods and both data sets, to test the spatial characteristics of physical proximity, isolation, and smallness for understanding tourism business links. Two small, isolated locations are surveyed: (i) Surselva-Gotthard in the Swiss Alps comprising the three communities Andermatt, Disentis and Sedrun (the area surrounding Sedrun is sometimes referred to as Tujetsch), and (ii) Longyearbyen on Svalbard in the Norwegian Arctic. In Surselva-Gotthard, the interviews helped to inform the role of geographic structure in the business network and in the isolation of peripheral actors identified in the SNA. In Longyearbyen, the interviews revealed weak and diffuse informal ties that were not explicitly identified in the quantitative network. Together, quantitative network analysis from simple survey data, combined with targeted follow-up interviews based on those results, helped to identify regionally specific opportunities and challenges for more effective governance.

## Social Network Analysis (SNA) and Proximity

SNA provides useful formal tools, qualitative and quantitative, for characterising networks of individuals or collectives (such as governments or businesses) and the strength and distribution of links within those networks [2], [10], [12], [13], [14], [15], [16], [17] examples of which were given above. One goal is to infer from the network structure important aspects of community dynamics, such as how actors and groups of actors (clusters or sub-groups) influence one another and how the entire community responds to external changes. Different types of links lead to different potentials for governance within and external to that network [18]; for instance, adjusting to social or environmental change. The direction and strength of communication links can also be used as a further indicator of power relations and influence [19].

Despite the extensive literature on SNA and the extensive literature on spatial characteristics of networks in innovation studies [20], [21] these two areas have the potential to be joined more [10]. SNA studies do not always explore spatial characteristics of networks, such as the network’s level of isolation from other networks or how the physical proximity of actors in a network might affect their interactions. An example exists where it has been done for tourism businesses [3], [4] as well as, from a different field, comparing SNA with other quantitative models to explain genetic diversity of southwest Pacific islanders [22]. Similarly, qualitative SNA data from tourism businesses have been used to validate quantitative network data [23] with similar approaches taken for other case studies [24], [25].

Yet many studies often pick a qualitative approach or a quantitative approach, rather than combining qualitative and quantitative methods in order to evaluate the level of information which SNA can and cannot provide [26]. An exception is [27] using SNA and other methods, quantitative and qualitative, to analyse psychologically one child’s experience of school-related places. Quantitative and qualitative methods should not be seen as a dichotomy, but as mutually complementary approaches giving different understandings of a phenomenon [10], [27].

When examining business networks, the evidence is mixed regarding the influence of (i) physical proximity of network actors and (ii) networks on interactions amongst the businesses in the network [28], [29]. For example, information and communication technologies (ICTs), including the internet, blur perceived proximity if these technologies are used [30] but that does not necessarily obviate geographical proximity effects [31]. Meanwhile, studies [32], [33], [34], [35], [36] indicate the challenges of formulating generic conclusions about how physical proximity and isolation impact business links. [20] summarises much of the literature in a useful typology, distinguishing five proximity factors relevant for links: 1. cognitive, 2. organizational, 3. social, 4. institutional, and 5. geographic referring to travel distance or travel time. An example of the application and extension of this typology is for knowledge networks [37].

When examining the fifth factor, geographic proximity, flows of information tend to decay with increasing distance meaning that information about the availability, suitability, and reliability of potential links decreases in quality and quantity [38], [39], [40]. Consequently, this tradition of research predicts that patterns of links are strongly driven by the geographic distribution of individuals and organizations as well as the ease with which they can exchange knowledge at different distances.

Nevertheless, neither the geographic distribution nor the ease of knowledge exchange is necessarily prominent in any case study. [41] examined the use of social media for organising the Occupy Wall Street movement and still found that increased geographical proximity increased links despite the ease of using the technology. [42] examined networks of inventors for German biotechnology concluding that technological development lessened the impact of geographical proximity on links because, over time, links formed with partners of partners, increasing the geographical distance of links. The wide diversity of case studies could be expected to yield the disparate results observed, supporting the relevance of the comparative analysis enacted for this paper.

## Case Study Overview

The Surselva-Gotthard area in central Switzerland comprises three main municipalities across two cantons, covering 525 km^2^, with the lowest point at the Rhine River (962 m) and the highest point at the peak of Piz Russein (3,640 m). The area has a resident population of 6,833 as of 2012, plus a substantial number of seasonal residents during the peak months of the winter and summer tourism seasons [43] (Table 1). Tourism businesses are generally small, numbering almost 170 depending on the exact definition of a tourism business. An exact census of tourism businesses does not exist, especially since many residents draw equally upon tourists and locals for their livelihood, often across several jobs.

**Table 1:** The case studies’ geographic and political characteristics.

Region	Surselva-Gotthard			Spitsbergen
Location	Switzerland, Canton Uri	Switzerland, Canton Grisons		Norway, Arctic Archipelago of Svalbard
Compared communities	Andermatt	Tujetsch (Sedrun)	Disentis	Longyearbyen
Surface area (km^2^)	62.16	13.99	91.07	37,673
Number of resident population	1,279	1693	2,067	2,495
Population per km^2^	20.58	121.02	22.70	0.07
Minimum elevation	1,360	1,230	969	sea level
Maximum elevation	3,001	3,327	3,614	1,713
Official administrative language	German, Romanic (only Tujetsch and Disentis)			Norwegian
Administrative centre	Disentis/Muster (1,130 m)			Longyearbyen (sea level)
Nationalities of resident population	79% Swiss	74% Swiss	89% Swiss	>30 different nations
Annual average precipitation (mm)	1,697	1,212	same station as Disentis	190
Economic dependency on tourism	75–95%	75–95%	75–95%	>30%, increasing
Other industry sectors	Military Services (in decline)	Hydro Power Generation	Administration, Education	Coal mining, Research

The region’s tourism sector is currently in flux. After a decade of decreasing guest numbers [44], a major development project called “Andermatt Swiss Alps” has been creating a new situation, with a shift in the regional power structures and introducing economic and environmental challenges and opportunities. The main identified threat currently is that the future development of Surselva’s tourism sector is linked too strongly to this large-scale project, leading to envy and tensions in the region [45].

The SNA conducted in Surselva-Gotthard covered 170 businesses of which 71 (42%) responded to the survey naming a total of 159 businesses as being within their network [3], [4]. The main locations of the businesses named were in the towns of Andermatt (52 businesses), Sedrun (50 businesses), and Disentis (31 businesses) which lie from west to east on the same road, while the Oberalp mountain pass between Andermatt and Sedrun is both a geographic and a political border between the two cantons. Additionally, 26 tourism related businesses came from the region outside these three communities. Then, informed by the SNA, one-on-one semi-structured interviews were completed with 20 actors from the cores and the peripheries of the networks indicated by high, medium, and low betweenness centrality—an SNA parameter describing the importance an actor has in linking with others [46]. For the comparative analysis in this paper, the businesses from outside the three towns and their linkages are exempted from the Surselva-Gotthard sample (Table 2).

**Table 2:** The SNA metrics of the case sites used and compared in this study. The mean proportion of inter-cluster links is higher in Longyearbyen than in Surselva-Gotthard, indicating lower modularity in Longyearbyen, the same as the modularity values directly show.

	Surselva-Gotthard	Andermatt	Sedrun	Disentis	Longyearbyen
Nodes	133	52	50	31	61
Links	1,420	259	448	176	206
Average Links per Node	10.89	4.98	8.96	5.67	3.377
Clustering Coefficient	0.453	0.571	0.51	0.485	0.309
Connectance	0.16	0.20	0.37	0.38	0.11
Modularity	0.337	0.138	0.173	0.116	0.287
Average Path Length	2.12	1.84	1.69	1.77	2.13

Svalbard is an archipelago in the high Arctic, 800 kilometres north of mainland Norway. Norway has sovereignty over the islands, but other countries have resource access rights through the Svalbard Treaty [47]. Longyearbyen is Svalbard’s main settlement, situated at 78°N with a population of about 2,500 as of 2012, approximately three quarters of whom are Norwegian with the rest coming from about three dozen countries, but mainly Thailand, Sweden, and Russia [48]. The population turnover rate is approximately 25% each year and the main industries are mining, higher education, research, and tourism [48]. No indigenous community preceded settlement. Residents are defined as those living there for more than six months, but by law they are only temporary residents, because they must retain a fixed address outside of Svalbard (Table 1).

85 businesses in Longyearbyen were identified as being in the tourism sector. They are predominantly owned and operated by Norwegians, with the owner-operators focusing on a steady cash flow (even if seasonal), but having minimal financial contingency and limited strategic business plans. They are not always entirely profit-driven, instead enjoying the lifestyle of independent working, which enables sacrificing business time for leisure and family time [49], [50].

The SNA in Svalbard covered all 85 businesses of which 21 (24.7%) responded to the survey, naming a total of 61 businesses as being within their network [51] (Table 2). The SNA subsequently informed 20 one-on-one semi-structured interviews completed with actors from the core and the periphery of the network, indicated by high, medium and low betweenness centrality.

In both cases, the tourism-related businesses and organisations comprise the nodes of the networks, while the links are formal and informal business collaborations. Links were generated based on printed questionnaires asking the responding businesses with which other businesses they professionally collaborate. Further questions on the topics and quality of such linkages were included in the surveys, but these data are not the subject of this paper’s analysis which focuses on the quantity and existence (or otherwise) of links. In the qualitative one-on-one interviews, the results of the survey were validated and discussed. The network graphs were presented to the interviewees at a later stage in the interviews and discussed in regard to their own perceived or expected network position.

For the data collection in Svalbard, the approving body for human subjects research is the Norwegian Social Science Data Services. For the data collection in Surselva-Gotthard, the approving body for human subjects research is the Working Group of the Swiss Ethical Committees for Research with Human Subjects (WGEC). Both bodies acknowledged that oral consent, recorded as part of each interview, is acceptable. No personally identifiable data were collected in either case study.

## SNA and Interviews in Surselva-Gotthard

[4] report the SNA for Surselva-Gotthard and [51] report the SNA for Longyearbyen. A comparison shows that the Surselva-Gotthard network compared to the Longyearbyen network has a more cohesive (higher link density) and more centralized structure with a strongly linked core of actors. The network of Longyearbyen is less densely linked and more compartmentalized or ‘modular’ than Surselva-Gotthard and its three towns, without a clear core-periphery separation. Both higher modularity and lower mean proportion of inter-cluster links in Longyearbyen than in the Surselva-Gotthard towns indicate this higher compartmentalization (Table 2). These structural patterns in formal ties suggest that Surselva-Gotthard may have a higher potential for quickly steering governance processes and actions with faster information flows [46], [52], [53], but may suffer from low diversity of new ideas and an uneven power distribution that may marginalise the opinions of peripheral actors and thus suppress new ideas [46], [52]. Conversely, Longyearbyen may have greater potential for new idea generation internally due to higher group diversity [46], [52], but less potential for community-wide fast governance intervention [4], [53].

**Fig 1:**
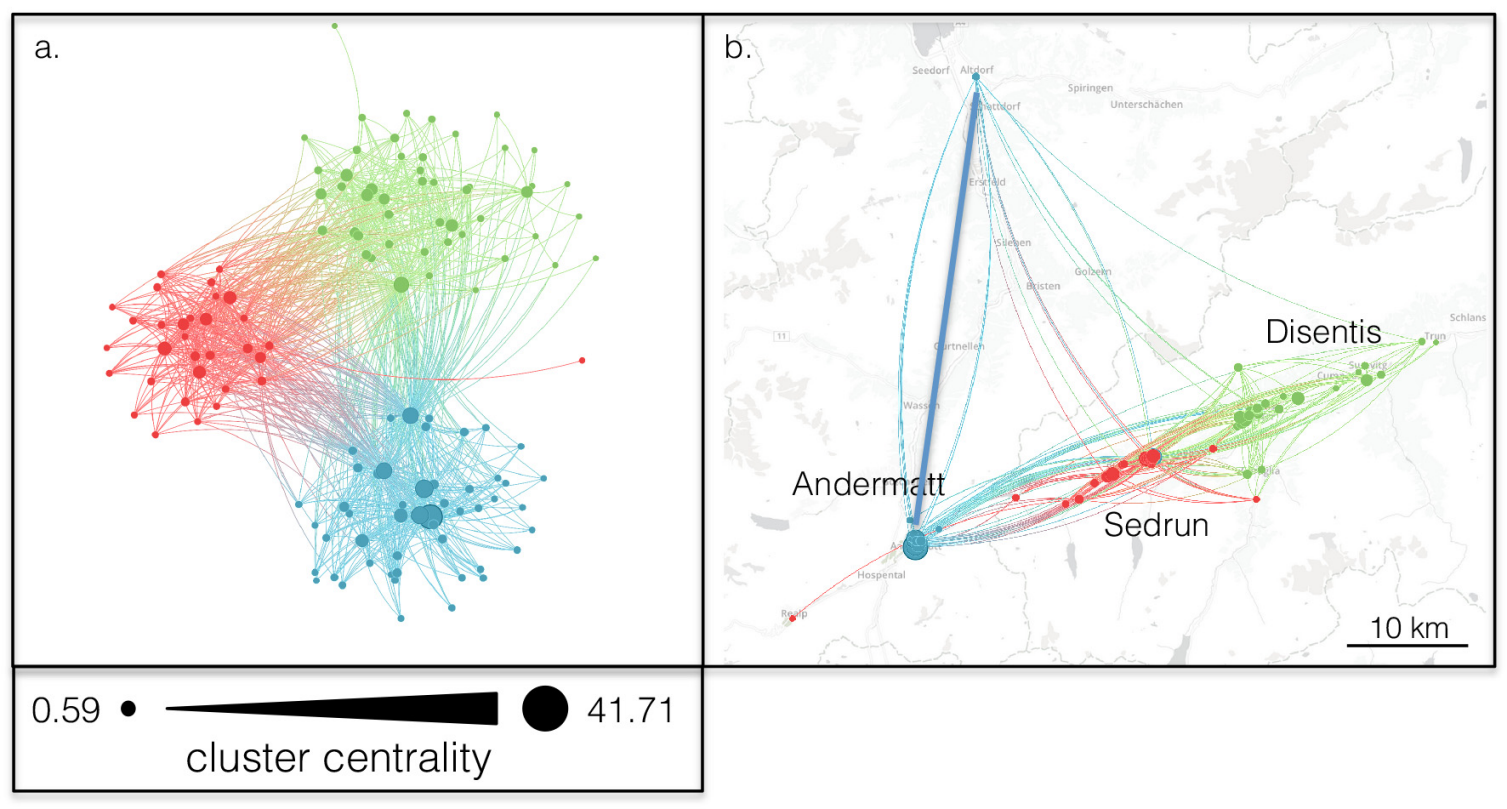
The Surselva-Gotthard tourism business collaboration network displayed in two ways: a) Force-directed layout where nodes that are more connected to one another cluster together in space, and b) geo-located in the three towns Andermatt, Disentis, and Sedrun. Each node is a business actor. Lines indicate self-described collaborative links between actors. Colour indicates clusters of actors that tend to collaborate more with one another than with those in other groups [56]. Modularity values are 0.33 (region), 0.14 (Andermatt), 0.12 (Disentis), and 0.17 (Sedrun). Collaboration clusters tend to be associated with geographic proximity. Nodes are sized by Cluster Centrality (c_*i*_), and the density distribution of c_*i*_ is indicated in the grey bar. *c*_*i*_ = (*l*_*i*_—*i*_*i*_) / (1 + *H*_*i*_) where: *H_i_* = −∑_*j*_*p_ij_* ln *p_ij_*; p^*ij*^ = l_*ij*_/l_*i*_; l_*i*_ = number of links of node *i* (i.e., 'degree'); l_*ij*_ = number of *i*'s links to cluster *j*; and i_*i*_ = number of links to nodes of a different cluster. A node is central to a cluster if it is highly connected and most of its connections are within its own cluster (as opposed to a different cluster). Visualised with VibrantData (http://vibrantdata.io.

**Fig 2:**
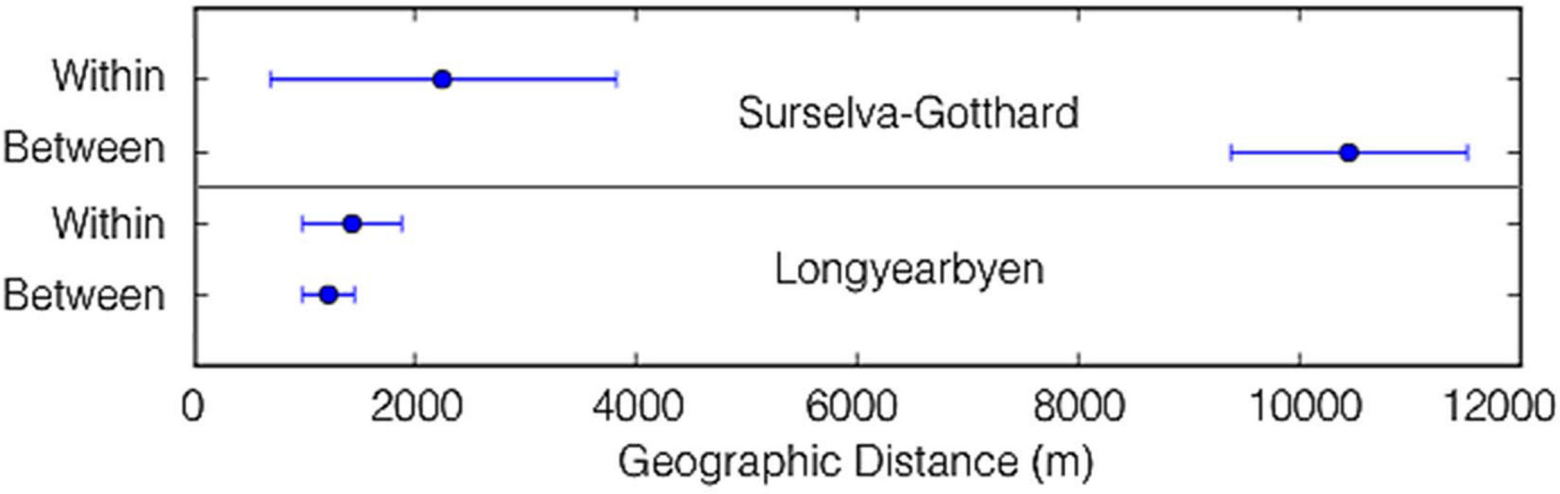
The relationship between geographic distance and collaboration modules for both the Surselva-Gotthard region in the Alps and Longyearbyen in the Arctic. The data presented are the mean and standard deviation of geographic distances among all pairs of businesses within vs between each collaboration module identified in Fig 1.

None of these SNA metrics displays the spatial location of the businesses. Fig 1 presents the SNA according to geo-location of the businesses in the three communities of Surselva-Gotthard. This network is characterized by three ‘modules’ [51] or groups of nodes (businesses) that tend to be linked to (i.e., collaborate with) one another more than to other nodes (Fig 1: coloured groups). These collaboration modules are significantly associated with geography: the mean geographic distance among all pairs of businesses within modules is significantly shorter than the mean geographic distance among all pairs of businesses between modules (Fig 2) This pattern is confirmed in that the ratio of links between businesses is highest within each town. For Andermatt’s links, 50% are internal; for Sedrun’s links, 52% are internal; and for Disentis’ links, 60% are internal. As well, links between towns are highest with adjacent towns: Andermatt-Sedrun has 26% of all links, Sedrun-Disentis has 28.2% of all links, and Andermatt-Disentis has only 5.7% of all links. This evidence gives a correlation between propinquity and links, supporting findings from other studies [54], [55]. The evidence does not prove causation (see also [40]), in terms of either propinquity causing links (which would be expected since people often prioritise those physical closest to them) or vice versa (which could happen if existing links cause businesses to move closer to each other).

Nonetheless, the Surselva-Gotthard interviews then provided possible causative mechanisms for propinquity leading to links, confirming the importance of physical proximity for business links and explaining why. The businesses usually had websites, but did not use ICTs extensively for their operations, with the website often being little more than a business card. Because most businesses were owner-operated, or employed a small number of staff, the owners spent their time on operational tasks, such as managing the property, dealing with clients, getting supplies, and accounting. Little time remained for networking outside of immediate, operational needs. It is easiest to work with those who are closest, rather than using email, Twitter, and/or Skype to forge and maintain networks with people who are farther away—especially if the links are for products, supplies, and on-site services rather than for knowledge or advice. This explanation supports the contention that ICTs will not necessarily undermine the geographic proximity effect [31]. Nonetheless, in the tourism service industry, significant cooperation can involve appointing others to deliver a service at a certain time and location, or by sending customers to each other, which ICTs can facilitate. Little such cooperation, however, was observed in Surselva-Gotthard.

Causative mechanisms for the uneven distribution of links amongst the three towns are also not evident from the quantitative SNA, but the interviews suggest possible factors. First, the main language in Andermatt is German compared to Romansh for Sedrun and Disentis. Second, Andermatt is in a different canton with a different cantonal government from the other two, a factor relevant for institutional and social proximity described by [20]. Third, the three towns are on the same road, but Andermatt is reached by a winding road which rises 800 m from Disentis to the Oberalp Pass (2,044 m above sea level), accessible only by train in the winter, before descending 600 m to Andermatt. These factors were raised in the interviews, are corroborated by the location’s physical geography, and explain causes for the lack of links between Disentis and Andermatt. The interviews further hinted at jealousy from historic rivalry, development patterns separating the locations, and the large ongoing investment in Andermatt. The reasons for limited links between Sedrun and Disentis, as hinted at in the interviews, were physical proximity and jealousy because of Sedrun recently seeking closer links with Andermatt due to the latter’s new development. Interpreting via social embeddedness [57], Sedrun and Andermatt’s links are being enhanced by social embeddedness while links between Sedrun and Disentis are being limited due to lack of social embeddedness.

In effect, the quantitative SNA indicates the possibility that the spatial layout of the Surselva-Gotthard towns might lead to some degree of town-based isolation, so the businesses focus their links on who is closest to them. That is not necessarily bad, especially if it is cheaper or more convenient to acquire and monitor products and services from nearer suppliers. The interviews confirm spatial layout as an important factor, in tandem with historical, political, personal, and industry-specific factors, including jealousy leading to the avoidance of links with rivals from other towns. The qualitatively gleaned possible causations (from the interviews) could explain the correlation (from the quantitative SNA). Thus, the qualitative interviews and SNA have complemented each other for understanding spatial characteristics of the tourism business networks and links.

A potential test for the influence of spatial layout on links is emerging. The Surselva-Gotthard region is planning a Destination Management Organisation (DMO) aiming to improve links within the tourism network, a strategy supported by the literature [58]. If inter-town links increase, then further evidence might emerge that a DMO can potentially overcome physical proximity—and other barriers—for forming links.

## SNA and Interviews in Longyearbyen

**Fig 3:**
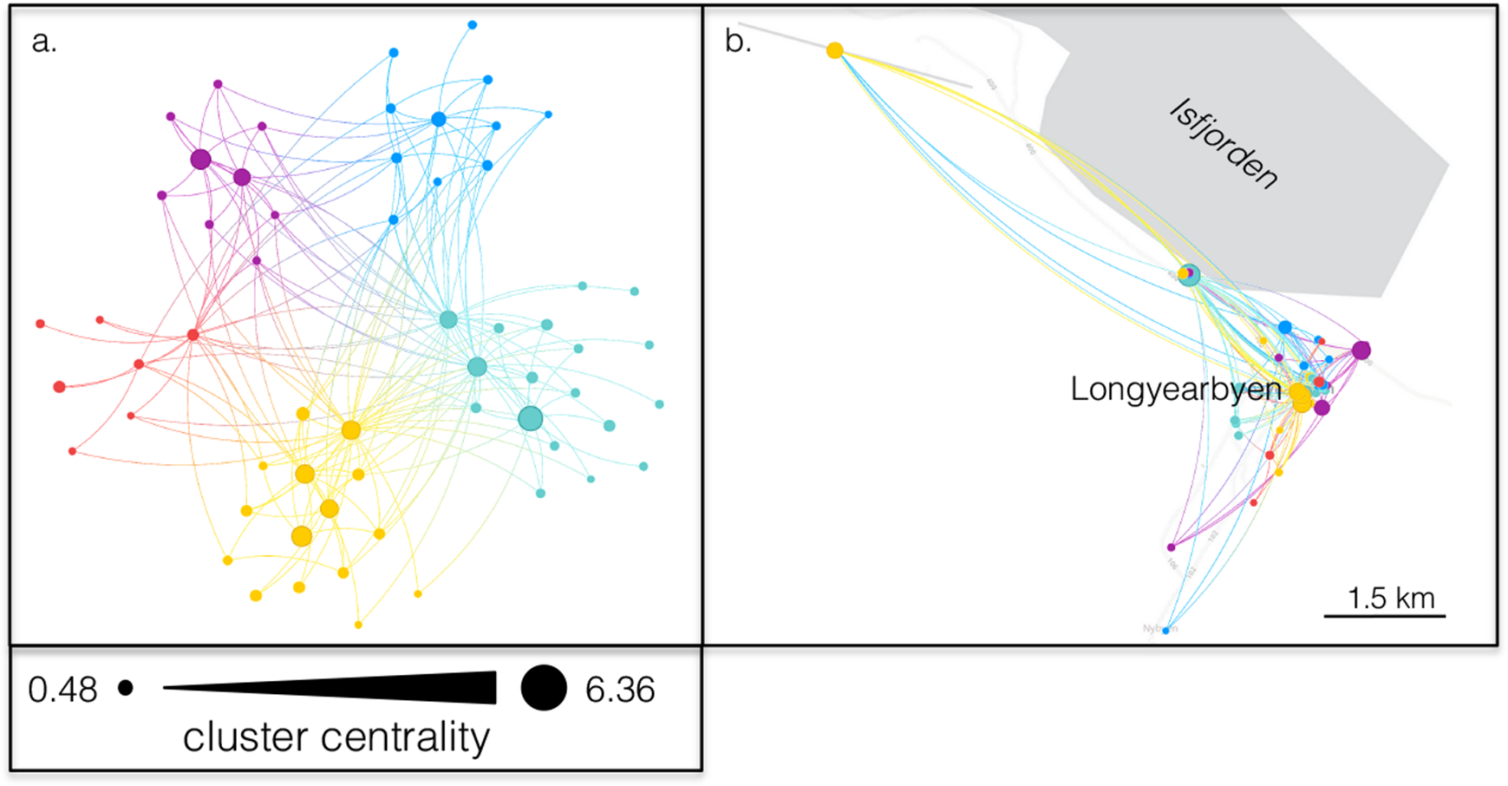
The Longyearbyen tourism business collaboration network displayed in two ways: a) Force-directed layout where nodes that are more connected to one another cluster together in space, and b) geo-located within the town of Longyearbyen. This network segments into five distinct collaboration clusters that appear to be independent of geographic distance. Modularity value is 0.29. Nodes are sized by Cluster Centrality. See Fig 1 for more details about cluster detection and node sizing.

Compared to the Surselva-Gotthard towns, the Longyearbyen SNA [51] exhibits higher compartmentalization of businesses. Five small clusters/subgroups are formed (Fig 3). These clusters emerge despite close physical proximity amongst the businesses (Fig 2), much closer than Surselva-Gotthard, and despite Longyearbyen businesses being much farther away from external links than Surselva-Gotthard businesses. The higher compartmentalization with more subgroups that are not defined by municipal boundaries (as in Surselva-Gotthard) may support the generation of new ideas especially through weak links between the subgroups [11]. The high population turnover rate and the internationalization also need to be considered as factors bringing a high rate of new strategies, products, and services to the tourism industry.

The isolation of Longyearbyen is further highlighted as explaining the SNA results [51], but the community’s isolation is an explanatory factor for some of the results—as confirmed by the qualitative interviews—rather than emerging from the quantitative SNA data. Isolation as an explanatory factor is further emphasised by Surselva-Gotthard having much less pronounced isolation and a much more externally connected tourism supply chain than Longyearbyen.

The qualitative interview data point to several geographic characteristics of the business links, since the smallness and isolation of the community were emphasised in the interviews. High competition and high fluctuation of businesses and business ownership were other reasons identified in the interviews which explain the SNA results of high modularity (compartmentalization) and low overall link density. The latter occurs despite the physical proximity of the businesses which the quantitative SNA identified but could not explain. Additionally, no ownership possibilities for land on Svalbard enhances a lack of place attachment and of community feeling, thus reducing interest in forging links [51].

The Longyearbyen interviews further indicate that the SNA results highlighted formal, direct business links and not broader, indirect business synergies. For example, the isolation and small size of Longyearbyen mean that if one business attracts tourists, then everyone potentially benefits, even if they do not have links [59]. This type of indirect facilitation among competing businesses occurs when one business increases the size of the tourist ‘pie’ which is then shared by all. That is, the interviews suggest that Longyearbyen’s spatial characteristics generate a feeling that enough business from tourists is available for everyone, so little anxiety exists about competition. The business environment was described as being highly competitive, but not cutthroat since there was always enough business to go around.

To most Longyearbyen respondents, links and mutual help were simply “business as usual” because small, isolated communities breed tightness and a community spirit through helping each other—as is often noted for island communities [60], [61] including for Longyearbyen [59] and, as well, for social groups more generally [62]. If one business could not serve a tourist, then the tourist would be passed onto another business. To the respondents, that is basic courtesy and necessity when living in a small, isolated location. Similarly, when a large cruise ship arrives with hundreds or thousands of tourists disembarking, the businesses know that they can only handle this situation together. An added layer is that many businesses have the same owner(s), so competition amongst those is reduced and clustering might be enhanced.

Consequently, the interviews revealed informal day-to-day links and indirect business facilitations that characterise this small, isolated community, while the quantitative SNA appears to have elucidated the formal business structure which is less tightly knit. Again, the methods complement each other for interpreting geographic characteristics influencing tourism business networks and links. In fact, advice and conclusions from previous literature [10], [26], [27] is confirmed that quantitative and qualitative methods should not be seen as a dichotomy, but instead as mutually complementary approaches giving different understandings of a phenomenon, in this case tourism business links.

## Geographic Characteristics from Each Method

In Surselva-Gotthard, the division of the regional case study into three towns appeared to strongly influence the business links and network structure (Fig 2). Longyearbyen, on the other hand, revealed collaboration clusters that were not associated with geospatial structure (Figs 2 and 3). Both case studies revealed how qualitative interviews drawing out geographic characteristics and quantitative SNA drawing out network characteristics can complement each other for understanding how geographic characteristics affect business links. In Surselva-Gotthard, the interviews interpreted the role of geographic structure in the business network and in the isolation of peripheral actors identified in the SNA. In Longyearbyen, the interviews revealed weak and diffuse informal ties that were not explicitly identified in the quantitative network. Those ties are related to the community’s smallness and isolation. Simultaneously, the sparse and modular structure of the quantitative network of business links suggests that the qualitative perception amongst actors of a collaborative business environment may be over-stated.

From the two methods, for Longyearbyen, high population and business turnover alongside compartmentalization of the community into subgroups may support the internal development and application of diverse and new ideas [46], [52]. That comes at a cost of less coordinated planning and reduced steering of collective action [52], and the preference for short-term visions and actions which could be at odds with longer-term interests and approaches, such as environmental and heritage conservation. In Surselva-Gotthard, a strong sense of place and cultural identity coupled with an efficient, centralized communication structure seems to empower links supporting longer-term visions. This tight social structure may incur costs of ‘groupthink’ [63] if it limits infusion or acceptance of new strategies, products, and services; that is, a tight social structure can dampen down suggestions of trying out different approaches because it has not been done before or because an individual is in the minority.

Even though isolation and smallness characterise both case studies, they manifested differently in the analyses. Surselva-Gotthard’s actors, spread across three towns within the case study site, displayed isolation from each other in the network, which was then corroborated by the qualitative interviews. Quantitative SNA captured the isolation of the towns from each other to some degree while qualitative interviews confirmed this result and provided reasons for the isolation. These findings support similar conclusions such as [62] that clustering rather than geographical tightness occurs when social groups are above 30 members, given that the Surselva-Gotthard case study had more than 30 members and displayed clustering in each town.

The interview responses from Longyearbyen indicated that isolation from the outside world significantly supports informal business links, a characteristic which, in this instance, the quantitative SNA did not immediately detect. In both case studies, the lack of corroboration by the case studies that technological development makes it easier to link with those at larger distances [42] likely occurred because, as [42] highlights, the German biotechnology inventor networks were heavily knowledge-based, whereas the tourism businesses in Surselva-Gotthard and Longyearbyen use their links more for products and services, rather than for knowledge exchange. The same explanation applies to this study not providing evidence to support the approach discussed in [37] because their focus is also on knowledge networking.

## Limitations and Further Research

A single SNA is frequently used to provide a snapshot of a network in space and time, with the spatial boundaries defined prior to the analysis and the temporal boundaries being the time period over which the survey is conducted. In Longyearbyen, many operators stay for only three-to-five years. Surselva-Gotthard’s tourism businesses were more stable, but still with an average time in business of only around a decade [51]. The SNA for this study was not designed to capture such short-term fluctuations nor to indicate the differences in links which could result due to different time scales for business operation.

While SNA is sometimes limited to a snapshot in time—but not always, as shown by analyses of Hungarian businesses from 1987–2001 [64], [65]—qualitative interviews can more readily point to longer-term trends—as long as those trends are within the respondents’ awareness and experience. The more stable population of Surselva-Gotthard can indicate more about changes over time than the highly mobile population of Longyearbyen. Similarly, the pre-defined spatial delineation of the research necessarily leaves out some businesses, such as shipping companies for Longyearbyen’s tourism since those companies are based outside of Svalbard. Again, the qualitative interviews can provide insights about links with businesses outside the SNA’s spatial and temporal boundaries. Limitations of interviews include the respondents’ biases, memories, and perceptions which are important for their own sake but which can skew an external researcher’s analysis [66], [67].

Quantitative SNA thus becomes useful through its potential to detect metrics or to hint at spatial and temporal characteristics which then trigger qualitative research for elaborating them. SNA also identifies relevant actors for interviews, given their roles in the network and their existing links, thereby providing a systematic baseline for moving forward with qualitative interviews which reveal further insights about the locations’ geographic characteristics. As with other studies on proximity effects, the work here does not directly distinguish between levels of action, such as individuals, social networks, firms, and markets [57] nor does it necessarily distinguish amongst different types of links.

Some of the first-order limitations of SNA in describing geographic characteristics can be overcome [20], [21], [68], [69]. Based on one round of SNA, spatial boundaries can be changed if the actors indicate links with businesses outside the originally delineated region, as was experienced in this study—which would support further analysis of how physical proximity, isolation, and smallness influence network ties. Snowball sampling was compared with a full sample for SNA applied to Surselva-Gotthard and Longyearbyen [51]. They demonstrate how snowball sampling in Longyearbyen picked up the shipping companies as well as other businesses outside of Svalbard which were missed in the initial pre-defined spatial extent of the survey. Similarly, snowball sampling demonstrated how many businesses in both case study sites which do not serve tourists directly nonetheless exist because of the tourism industry, such as plumbers and grocery shops whose clientele relies to a large extent on tourist accommodation businesses. Further analyses could incorporate these businesses. Finally, repeating the SNA every few years for a longitudinal study would indicate changes over time.

## Conclusion

This study combined two methods in two case studies for understanding tourism business links particularly with regards to the role of physical proximity, isolation, and smallness. The qualitative interviews point to the influence of spatial characteristics that do not appear in the quantitative SNA for Longyearbyen, but which do appear to some degree for Surselva-Gotthard’s quantitative SNA. In both cases, quantitative SNA provided the initial insights which the qualitative interviews were then able to investigate. Combining methods and data yields the most comprehensive understanding of the identified geographical characteristics influencing the business networks and understanding their links with and influences on each other.

Running a tourism business in a small, isolated location has difficulties due to these spatial characteristics. Understanding how the spatial characteristics influence links, and potentially vice versa, yields advice for the businesses on improving their use of links with other businesses—which, as seen in Longyearbyen, does not necessarily entail diminishing a competitive spirit. Given the ongoing, rapid social and environmental changes affecting tourism destinations worldwide, enhancing local links might mean survival for many of the owner-operators.
